# Childhood adversity and psychotic‐like experiences in adolescence: Investigating a pathway via disrupted pubertal and grey matter development

**DOI:** 10.1002/jcv2.70159

**Published:** 2026-08-18

**Authors:** Megan Thomas, Sarah Whittle, Elizabeth M. Haris, Vanessa L. Cropley

**Affiliations:** ^1^ Orygen The National Centre of Excellence in Youth Mental Health Parkville Victoria Australia; ^2^ Centre for Youth Mental Health University of Melbourne Parkville Victoria Australia; ^3^ Melbourne School of Psychological Sciences University of Melbourne Parkville Victoria Australia; ^4^ School of Psychology Deakin University Burwood Victoria Australia

**Keywords:** adversity, neurodevelopment, psychosis, puberty

## Abstract

**Background:**

Childhood adversity increases psychosis risk, but the biological mechanisms responsible remain elusive. A possible but underexplored pathway involves altered pubertal and grey matter (GM) development.

**Methods:**

In youth from the Adolescent Brain Cognitive Development study (*N* = 3462), we investigated the effect of adversity at age 9–11 (indexing threat, socioeconomic deprivation and interpersonal deprivation) on psychotic‐like experiences (PLEs) four years later, and the mediating role of pubertal timing and tempo. We then investigated whether pathways could be further explained by the influence of puberty on longitudinal GM change. Analyses for males and females were conducted separately.

**Results:**

Associations were found between all adversity types and greater PLEs, of which most were mediated by earlier pubertal timing and/or slower tempo. Mediation effects tended to be stronger in females, and for socioeconomic deprivation pathways. Results did not support the presence of serial mediation pathways from adversity to PLEs via puberty‐related GM variation.

**Conclusion:**

Findings suggest that disruption to pubertal development may contribute to the link between adversity and psychosis risk, but not by altering GM development. Investigating puberty alongside alternative biological systems may help contextualise our findings.

## Key Points

**What's known?**

- Childhood adversity is associated with an increased risk for psychosis, but the biological mechanisms involved are unclear.
- Alterations in pubertal and related grey matter (GM) development may contribute to this relationship, but no previous studies have examined this.

**What's new?**

- Our findings revealed that variation in pubertal timing and tempo mediated associations between several adversity types and greater psychotic‐like experiences (PLEs), but not by influencing GM development.

**What's relevant?**

- Future work should investigate the role of other biological domains alongside puberty in these pathways.

## INTRODUCTION

Psychosis is widely thought to have neurodevelopmental underpinnings (Murray et al., 2017). Adolescence represents a key period for its emergence, with the peak of illness onset falling between age 15 and 25 (Patel et al., 2021), and subclinical symptoms arising several years beforehand (Nelson & McGorry, 2020). Longitudinal studies suggest that individuals who later develop psychosis exhibit accelerated GM loss in adolescence, particularly in the frontal, parietal, temporal and cingulate cortex (Merritt et al., 2021). These structural changes are thought to at least partly reflect the exaggerated pruning of synapses, and disruption in the balance of excitatory‐to‐inhibitory (E/I) neurotransmission (Hamilton et al., 2024; Howes & Onwordi, 2023).

Childhood adversity, an established risk factor for psychosis (Zhou et al., 2025), may influence these processes, with studies linking early‐life stress to altered synaptic pruning and E/I circuitry in animals (Caballero et al., 2021; Calcia et al., 2016), and GM development in humans (Teicher & Samson, 2016). Integrating these findings, Howes and Shatalina (2022) proposed that adversity and other risk factors interact throughout development to disrupt programing of synaptic refinement and E/I signalling. The resulting latent vulnerability purportedly interacts with subsequent stress to produce over‐pruning of excitatory cortical synapses in adolescence (reflected as GM loss). Our recent study revealed findings broadly consistent with this model (Thomas et al., 2025), with decline in frontoparietal GM partially mediating associations between childhood adversity and subclinical psychotic symptoms in early adolescence. Notably, this effect was only evident for certain adversity types, suggesting that pathways linking different exposures to psychosis risk may not be uniform.

Growing evidence suggests that different dimensions of adversity may confer risk for psychopathology through partially distinct neurobiological mechanisms. The dimensional model of adversity and psychopathology (DMAP) theorises that the impacts of threat (experiences involving harm) differ from those of deprivation (a lack of environmental input) (McLaughlin et al., 2014). Threat is proposed to sensitise fear learning circuits, with resulting alterations in fronto‐limbic function and structure. Deprivation is instead thought to affect synaptic proliferation and pruning, producing widespread GM reductions in the frontoparietal cortex. Since the DMAP's conception, research supporting this distinction has emerged (McLaughlin et al., 2019), including in the psychosis spectrum (Thomas et al., 2023). Further, studies have suggested that socioeconomic (e.g., low household income) and interpersonal (e.g., neglect) deprivation may also have diverging effects (Beck et al., 2024; Lawson et al., 2017; Thomas et al., 2025; Vannucci et al., 2023). It is therefore plausible that different forms of adversity act on psychosis risk pathways in distinct ways. However, models of psychosis aetiology typically consider psychosocial stress as one monolithic construct, and thus, divergency in these pathways has scarcely been investigated.

These models also fail to fully explain why symptoms tend to arise in adolescence, despite risk factors including adversity often originating much earlier. It has been proposed that pre‐existing vulnerabilities for psychosis (such as the aforementioned changes to synaptic pruning and E/I signalling) are unmasked by puberty, giving rise to altered neurodevelopmental trajectories that increase psychosis risk (Barendse et al., 2023). Puberty plays a key role in the sculpting of neural circuitry in adolescence (Pfeifer & Allen, 2021), with age‐independent associations demonstrated between pubertal maturation and GM loss in the frontal and parietal cortex (Vijayakumar et al., 2018, 2021)—regions implicated in both psychosis and childhood adversity. Individual variation in the timing (MacSweeney et al., 2023) and tempo (i.e., pace) (Vijayakumar et al., 2021) of puberty are also associated with GM variation in similar regions. Preclinical studies suggest that puberty‐related GM reductions may reflect synapse loss, and that puberty contributes to the maturation of inhibitory circuitry (Delevich et al., 2021). Furthermore, rodent models of schizophrenia suggest that many bio‐behavioural illness characteristics (e.g., dopamine dysregulation, neuroinflammation and elevated locomotor activity), induced by experimental insults (e.g., hippocampal legions or prenatal immune challenges), only emerge following pubertal onset (Gomes et al., 2016), supporting the notion that puberty unmasks these processes. Thus, it is plausible that puberty triggers psychosis risk mechanisms by initiating synaptic pruning and circuit refinement, which when combined with pre‐existing aberrations embedded by adversity, leads to atypical GM development.

As well as embedding vulnerabilities that are later unmasked by puberty, adversity may influence the features of puberty itself. For example, threat/trauma is consistently found to accelerate pubertal timing (Colich et al., 2020), particularly in females (Ho et al., 2024). Deprivation may also influence pubertal timing but findings are mixed (Colich et al., 2020, 2022; Ellis et al., 2009; Shaul et al., 2024). Moreover, early puberty has been found to predict greater psychopathology (Ullsperger & Nikolas, 2017), including, in recent work from the Adolescent Brain Cognitive Development (ABCD) cohort, psychotic symptoms (Damme et al., 2024; Larson, Chaku, et al., 2025). Emerging evidence has also linked adversity to altered pubertal tempo (Hamlat et al., 2022; Suglia et al., 2020), and, again in the ABCD cohort, tempo to psychotic symptoms (Larson, Chaku, et al., 2025). If, as proposed, puberty unmasks psychosis vulnerabilities embedded earlier in development, then adversity‐related changes to its timing/tempo might affect psychosis risk by influencing when/how these vulnerabilities emerge.

Given these findings, we used data from the ABCD study to test the hypothesis that childhood adversity leads to alterations in pubertal development, which in turn interact with pre‐existing vulnerabilities (including adversity‐related effects) to influence GM development and psychosis risk. We also investigated whether these mechanisms unfold in an adversity dimension‐specific manner. Childhood adversity was quantified using the dimensions of deprivation and threat, with deprivation further parsed into interpersonal (dep‐IP) and socioeconomic (dep‐SE) subtypes. Pubertal development was indexed using pubertal timing and tempo, and psychosis risk using PLEs, which, when experienced in youth, correlate with greater risk of developing psychotic disorders (Sullivan et al., 2020). As puberty involves distinct biological processes in males and females (e.g., sex‐specific hormonal changes and patterns of physical maturation), analyses for each sex were conducted separately.

Our first aim was to determine whether variation in pubertal timing and tempo mediated associations between threat, dep‐IP and dep‐SE in childhood (measured at age 9–11 years—around the start of puberty (Rosenfield et al., 2009; Tinggaard et al., 2012)), and PLEs four years later (when psychosis incidence begins to rise (Dalsgaard et al., 2020)). Our second aim was to investigate whether these pathways could be further explained by the influence of pubertal timing/tempo on GM development.

Based on the DMAP and associations in the previous literature, we hypothesised that all adversity measures would be associated with greater PLEs, but that mediation pathways would vary by dimension. For threat, we predicted mediation via earlier pubertal timing and steeper GM decline in the ventromedial prefrontal cortex (vmPFC). For dep‐SE, we predicted mediation through earlier or later puberty and steeper GM decline in frontoparietal regions. We predicted that dep‐IP pathways would not be mediated by puberty. Further, we hypothesised that females would demonstrate stronger mediation by early puberty in threat‐PLE pathways than males, given that associations between early puberty and both threat (Ho et al., 2024) and psychopathology (McGuire et al., 2019) have been more consistent in females than males in prior work. We also speculated that neural mediation effects for dep‐SE in females might extend to the cingulate and occipital cortex, considering our previous findings in this cohort (Thomas et al., 2025). Given limited research, we made no hypotheses regarding pubertal tempo.

## METHODS

This study was preregistered with the Open Science Framework (registration DOI: https://osf.io/zreup).

### Participants

Participants were sampled from the ongoing ABCD study (release 5.1), comprising 11,868 youth across 22 research sites in the Unites States (see Garavan et al. (2018) for sampling and recruitment procedures). Given the three‐level structure of the ABCD dataset (participants nested within family within site), and the lack of established methods for serial mediation analysis in three‐level data, 1 participant per family was randomly selected.

### Measures

#### Exposure to adversity

Following prior work (Berman et al., 2022), composite scores for adversity dimensions were created by aggregating several youth and caregiver‐rated measures from the first visit (i.e., baseline; age 9.8–12.3) (see Zucker et al. (2018) and Gonzalez et al. (2021) for measure collection details). Our threat construct included interpersonal trauma, neighbourhood crime, family conflict and bullying by peers; dep‐SE comprised insufficient material resources, low parental education and neighbourhood disadvantage; and dep‐IP indexed caregiver neglect. Full measure information and aggregation procedures are provided in Appendix S1.

#### Psychotic‐like experiences

PLEs at baseline and year 4 (age 12.6–15.8) were quantified using the Prodromal Questionnaire–Brief Child Version (PQ‐BC): a youth‐rated measure of subclinical psychotic symptoms demonstrating adequate construct validity, internal reliability and measurement invariance in the ABCD (Karcher et al., 2018, 2020). During a computerised assessment, participants were asked whether they had experienced 21 different PLEs in the past month. For each item, participants received a score of 0 (not endorsed), one (endorsed with no associated distress) or 2–6 (endorsed plus 1–5 for associated distress level). The sum of items denoted total PLE score (range 1:126).

#### Pubertal development

Pubertal development was measured annually (from baseline to year 4) using the pubertal development scale (PDS), a questionnaire probing perceptions of development in various pubertal domains (Petersen et al., 1988). Seven items were used to compute mean PDS scores at each timepoint, ranging from 1 (has not yet begun) to 4 (seems complete). Given frequent “I don't know” responses from youth in early timepoints, caregiver‐rated PDS scores were used for the first two timepoints, and youth‐rated for the subsequent three (see Ellis et al., 2011 and Ruttle et al., 2013 for similar mixed‐informant approaches).

### MRI acquisition/preprocessing

Imaging acquisition (Casey et al., 2018) and processing (Hagler et al., 2019) methods in the ABCD are described elsewhere. Briefly, T1‐weighted MPRAGE images (1 mm isotropic) were obtained from participants at baseline, year 2 and year 4 using 3 T (3T) magnetic resonance imaging (MRI). For scanners with the required functionality (26/29 at baseline), prospective motion correction was used to mitigate motion‐related artefacts. Pre‐processing of T1‐weighted images was conducted by the ABCD Data and Informatics Centre, and included a rigorous quality checking procedure, correction for artefacts and the extraction of structural metrics using FreeSurfer (version 5.3). For the present study, volume estimates for 34 cortical and 7 subcortical regions (per hemisphere), delineated using the Desikan‐Killiany (Desikan et al., 2006) and automated subcortical (Fischl et al., 2002) atlases, respectively, were obtained from the ABCD tabulated data. We chose to examine regions across the entire brain, given limited prior work examining pathways from adversity to psychopathology via puberty and neurodevelopment, and emerging evidence implicating a broader range of regions in adversity‐related neural effects than previously appreciated (Li et al., 2023; Rakesh & Whittle, 2021; Tymofiyeva et al., 2022). As recommended (Hagler et al., 2019), data failing the preprocessing quality check were excluded from analyses.

### Statistical analysis

#### Software

Statistical analyses were performed in *R* (version 2024.12.1) and Mplus (version 8.1). Exclusions and missing data handling procedures are outlined in Supporting Information S1: Appendix S2.

#### Estimating pubertal tempo and timing

Age and PDS scores at 5 timepoints were used to obtain subject‐specific estimates of pubertal timing and tempo. Logistic growth curves were used to model pubertal development, given work indicating that puberty follows an s‐shaped (sigmoidal) trajectory (Marceau et al., 2011), and guidelines for investigating puberty in censored datasets (Mendle et al., 2019). Logistic curves were fit using the following 4‐parameter growth function, where L and U represent the lower and upper asymptotes (fixed at 1 and 4, respectively, reflecting the start and end of puberty); $x_{0}$ is the x value (i.e., age) at the curve's inflection point; and k is the growth rate (curve steepness).

$$\text{PDS}\;\text{score}=L+\frac{U-L}{1+e^{-k\left(\text{age}-x_{0}\right)}}$$

Logistic models assume that the curve's steepest growth rate is at the point where it inflects, located halfway between the lower and upper asymptote (where PDS = 2.5). Individual variation in the x value at the inflection point ($x_{0}$) and the corresponding slope parameter (k) can thus be used to index variation in pubertal timing (i.e., age at mid‐puberty) and pubertal tempo (rate of development at mid‐puberty), respectively (Mendle et al., 2019). To derive fixed (average) and random (subject‐specific) estimates for these parameters, the above growth function was embedded within a nonlinear mixed effects model using the “nlme” *R* package, in males and females separately. Subject‐specific coefficients for $x_{0}$ and k were extracted from model output.

#### Estimating regional grey matter volume slope

Using 3 biannual timepoints, multilevel growth models were adopted to derive subject‐specific estimates for regional GM volume intercept (initial level) and slope (rate of change). In males and females separately, models of GM volume were fit for each region (averaged across hemispheres), including fixed effects of age (using the best fitting average trajectory ‐ linear or quadratic) and random effects for intercept and linear slopes to capture subject‐specific variation. Intercepts were fixed at the (sex‐specific) average age of participants at baseline. Random intercept and linear slope terms were then extracted for each region, denoting each subject's deviation from the average group (male or female) trajectory.

#### Primary analyses

**FIGURE 1:**
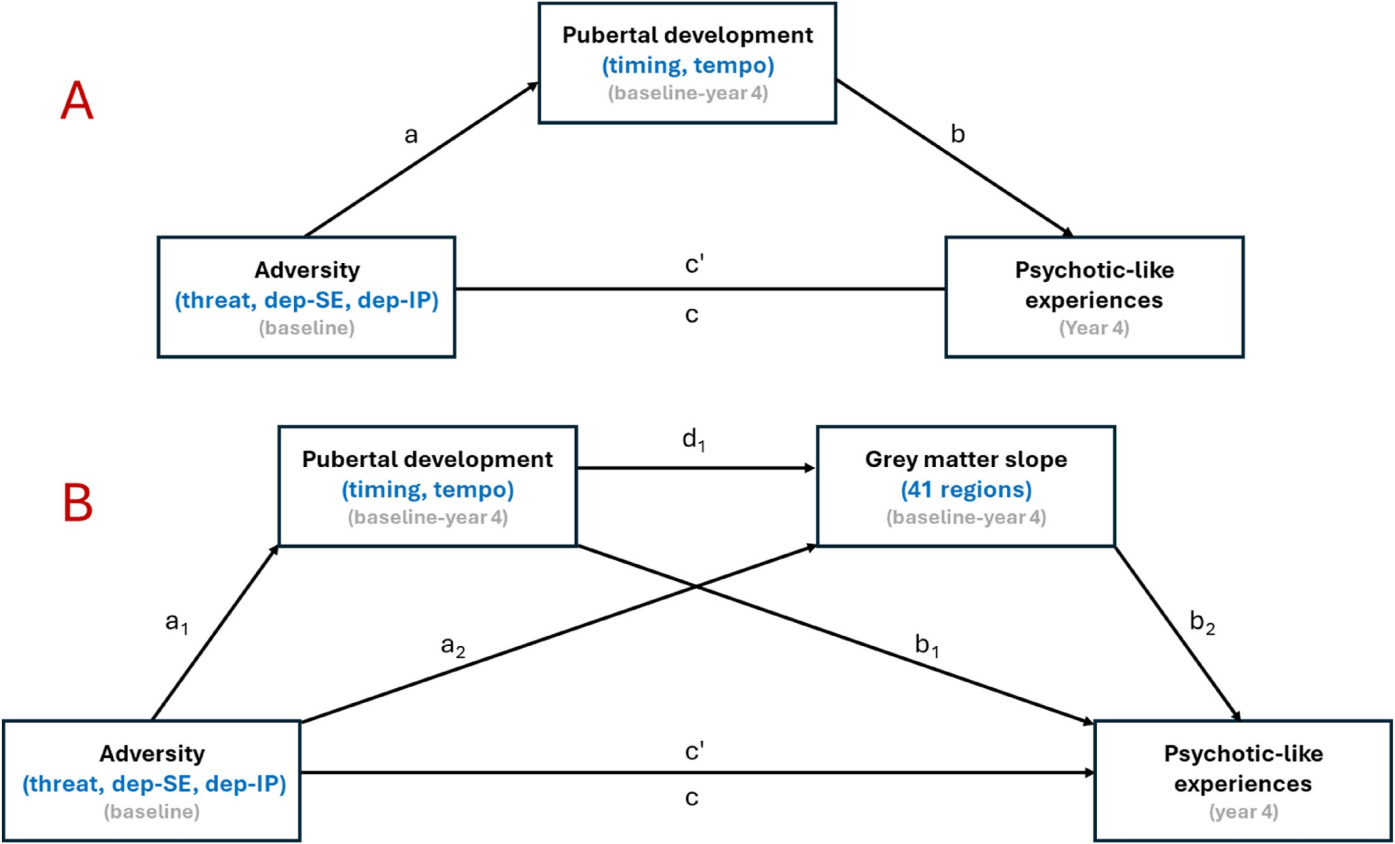
Mediation and serial mediation models. Panel A: mediation models investigating indirect effects from adversity to psychotic‐like experiences via pubertal development; Panel B: serial mediation models investigating indirect effects from adversity to psychotic‐like experiences via pubertal development *and* grey matter slope. Dep‐IP, interpersonal deprivation; Dep‐SE, socioeconomic deprivation.

A series of mediation and serial mediation models were conducted using Mplus (Figure 1). In all models, age at baseline and the time between baseline and year 4 were included as covariates; site was included as a clustering variable to account for subject nesting within site; and PLE scores were modelled as count variables with a negative binomial distribution. *p*‐values for indirect effects were derived using maximum likelihood estimation and bootstrapping (1000 reps) to generate robust standard errors.

First, mediation models were used to examine pathways from adversity constructs at baseline (threat, dep‐IP and dep‐SE) to PLE scores at year 4, via pubertal timing and pubertal tempo. Twelve models were performed in total (i.e., Three adversity types and 2 pubertal metrics = 6 models in males and females). False Discovery Rate (FDR) correction was applied across *p*‐values within each sex (significant at *p*_FDR_ < 0.05).

For pathways demonstrating significant mediation, serial mediation models were used to test for indirect effects from the relevant childhood adversity construct to PLEs via the relevant pubertal metric *and* regional GM volume slope, sequentially. Given our focus on adolescent GM change, all models included the corresponding random intercept term as a covariate. For each pathway investigated, FDR correction was applied across all 41 regions (*p*_FDR_ < 0.05).

#### Secondary analyses

As cortical volume is derived from thickness and surface area, which are influenced by different developmental processes (Tamnes et al., 2017) and may be distinctly affected by different adversity dimensions (Machlin et al., 2023), we repeated our serial mediation models for each metric in turn, applying the same multiple comparison correction.

#### Sensitivity analyses

In additional sensitivity analyses, significant models were repeated while controlling for (1) baseline body mass index (BMI), given associations between BMI and pubertal development (Chen et al., 2019) and childhood adversity (Lee et al., 2019); (2) baseline PLE score, to determine the specificity of effects to PLEs at year 4; and (3) all other adversity dimensions (simultaneously), as these exposures often cooccur.

## RESULTS

Following exclusions (see Supporting Information S1: Appendix S2), our final analytic sample comprised 3462 participants. Demographics for included and excluded participants are provided in Table 1 (total sample) and Supporting Information S1: Tables S1 and S2 (males and females separately). Correlations among non‐brain study variables are displayed in Supporting Information S1: Tables S3 and S4.

**TABLE 1:** Included and excluded sample characteristics at baseline.

	Included (*N* = 3462)		Excluded (*N* = 8406)				
	*M*	SD	*M*	SD	Test statistic	*p*	*d*
Age (months)	119.248	7.358	118.867	7.55	*t* (6602) = −2.547	0.011	−0.051
Income‐to‐needs ratio^a^	3.924	2.396	3.529	2.413	*t* (6102) = −7.785	<.001	−0.164
BMI (kg/m^2^)	18.669	4.053	18.896	4.325	*t* (6847) = 2.719	0.007	0.054
PQ‐BC score	5.979	10.057	6.458	10.827	*t* (6904) = 2.303	0.021	0.046
PDS score	1.557	0.452	1.621	0.498	*t* (7135) = 6.765	<.001	0.135

	***N***	**%**	***N***	**%**	**Test statistic**	***p***	***v***
Sex (male)	1862	0.538	4326	0.515	*X* ^2^ (1, *N* = 11,865) = 5.12	0.024	0.021
Race							
White	2788	80.5	6009	71.5	*X* ^2^ (1, *N* = 11,868) = 104.16	<.001	0.094
Black	506	14.6	2012	23.9	*X* ^2^ (1, *N* = 11,868) = 126.85	<.001	0.103
Asian	237	6.8	514	6.1	*X* ^2^ (1, *N* = 11,868) = 2.09	0.148	0.013
Native American	130	3.8	300	3.6	*X* ^2^ (1, *N* = 11,868) = 0.19	0.66	0.004
Pacific Islander	12	0.3	41	0.5	*X* ^2^ (1, *N* = 11,868) = 0.8	0.37	0.008
Other	200	5.8	600	7.1	*X* ^2^ (1, *N* = 11,868) = 7.01	0.008	0.024
None given	38	1.1	148	1.8	*X* ^2^ (1, *N* = 11,868) = 6.56	0.01	0.024

Abbreviations: BMI, body mass index; *d*, Cohen's *d*; *M*, mean; PDS, Pubertal Development Scale; PQ‐BC, youth‐rated Prodromal Questionnaire–Brief Child Version; SD, standard deviation; *v*, Cramer's *v*.

^a^ <1 = below poverty threshold.

### Puberty slopes

**FIGURE 2:**
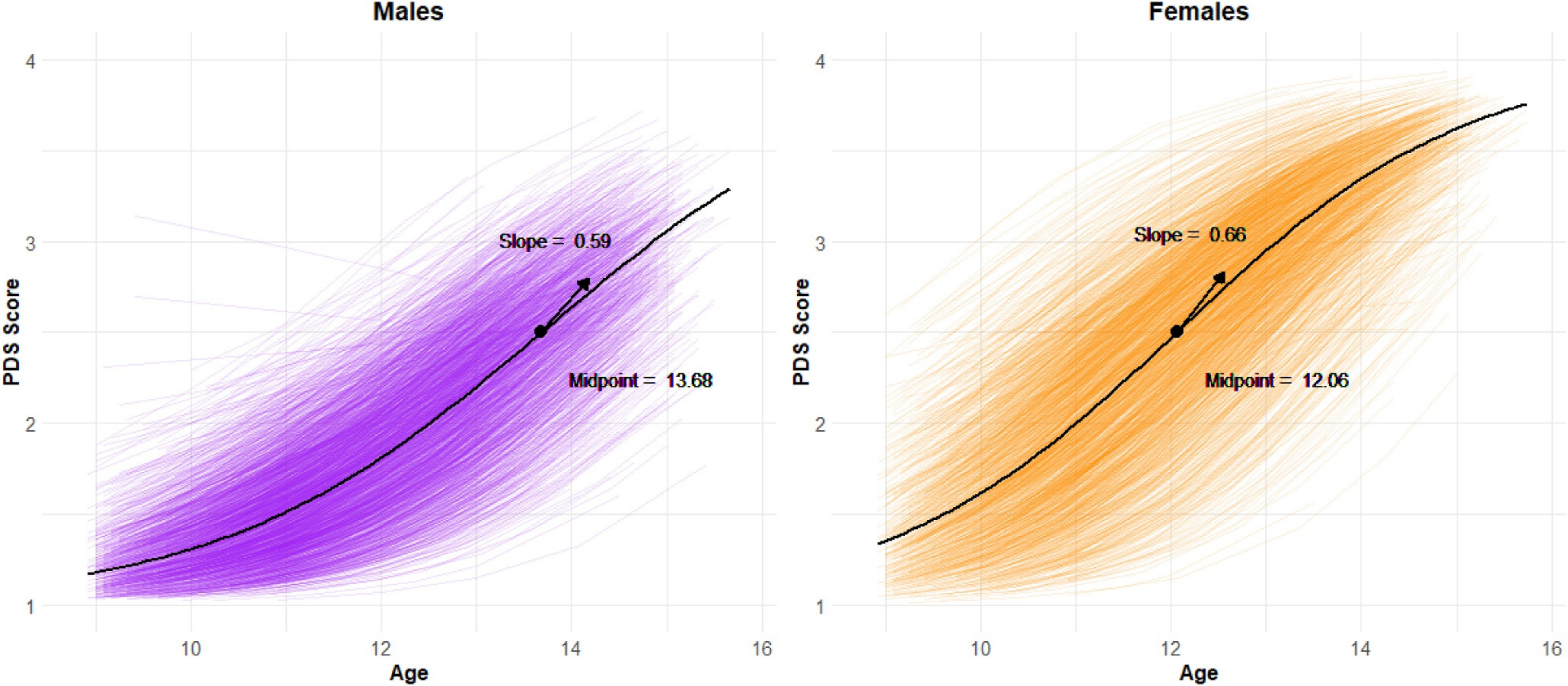
Estimated trajectories of pubertal development. PDS = pubertal development scale; midpoint = estimated age of mid‐puberty (i.e., when PDS score reaches 2.5); slope = estimated rate of change at mid‐puberty (i.e., the curve's inflection point); black lines = average estimated trajectory in males (left) and females (right); purple and orange lines = estimated trajectory for each subject.

Average (fixed) and subject‐specific (random) pubertal trajectories are displayed in Figure 2. The mean estimated age at mid‐puberty (indexing pubertal timing) was 13.68 in males and 12.06 in females. The average slope parameter at mid‐puberty (indexing pubertal tempo) was 0.59 in males and 0.66 in females. Age and slope at mid‐puberty were positively correlated in males (*r* = 0.27, *p* < 0.001) and females (*r* = 0.50, *p* < 0.001), indicating that youth undergoing puberty earlier tended to develop more slowly.

### Grey matter volume slopes

Of the 41 regions investigated, longitudinal GM volume change was best characterised by a linear trajectory in 6 (3 cortical, 3 subcortical), and a quadratic trajectory in 35 (31 cortical, 4 subcortical) in males. In females, all regions followed a quadratic trajectory (see Supporting Information S1: Figures S1–S4).

### Primary analyses

**FIGURE 3:**
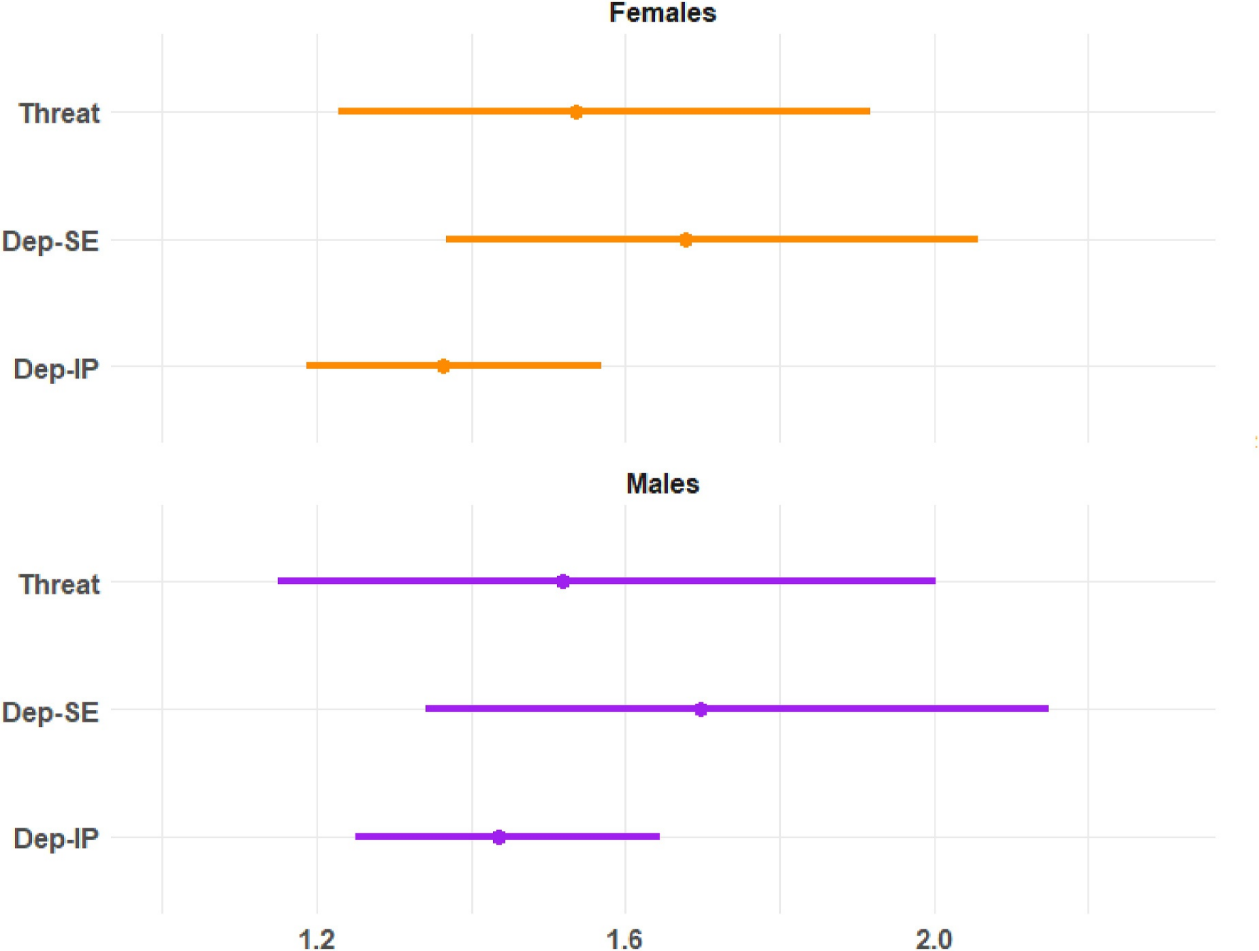
Effect of adversity dimensions on PLEs. Dep‐SE = socioeconomic deprivation; Dep‐IP = interpersonal deprivation; filled circles represent incidence rate ratios (IRR), lines represent IRR confidence intervals; all models included age at baseline and the time from baseline to year 4 as covariates.

**FIGURE 4:**
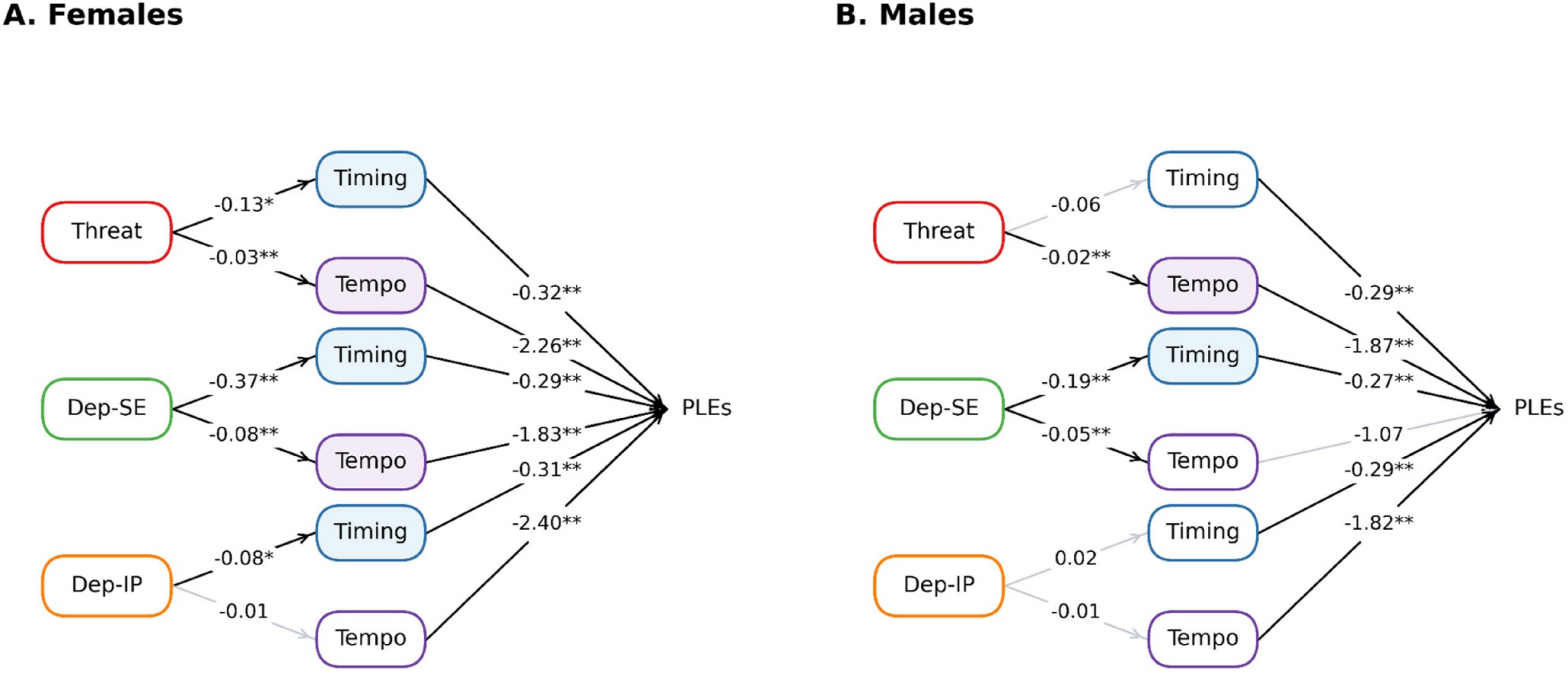
Mediation pathways from adversity dimensions to PLEs via pubertal timing and tempo. Dep‐SE = socioeconomic deprivation; Dep‐IP = interpersonal deprivation; PLEs = Psychotic‐like experiences; all values = unstandardised coefficients for paths a and b (see Figure 1A); * = *p* < 0.05; ** = *p* < 0.01; light grey lines = non‐significant; shaded pubertal mediators indicate that the indirect pathway reached significance following FDR correction; all models included age at baseline and the time from baseline to year 4 as covariates.

Significant total effects (i.e. Figure 1A, path c) were observed for all models. Specifically, all adversity types (at baseline) were associated with greater PLEs (at year 4) in males (threat: *p* = 0.003; dep‐SE: *p* < 0.001; dep‐IP: *p* < 0.001) and females (all *p* < 0.001), respectively (Figure 3). In females, with the exception of Dep‐IP and pubertal tempo, earlier pubertal timing and slower pubertal tempo significantly mediated associations between adversity constructs and PLEs, explaining between 8.5% (dep‐IP and pubertal timing) and 27.6% (dep‐SE and pubertal tempo) of the total effect. In males, significant mediation was found in two models: earlier pubertal timing mediated the effect of dep‐SE on PLEs (9.1%), and slower pubertal tempo mediated the effect of threat on PLEs (8.4%). No other indirect effects survived FDR correction (Table 2 and Figure 4).

**TABLE 2:** Indirect pathways from adversity to PLEs via puberty.

X	M	Y	Sex	a (*X* > M)	b (M > *Y*)	Direct^a^ (*X* > *Y*)	Indirect (*X* > M > *Y*)	% Total effect mediated	Indirect FDR adj. *p*
Dep‐SE	Age at mid‐puberty	PLEs	Females	−0.368**	−0.290**	0.448**	0.107**	19.265	**<.001**
Dep‐SE	Slope at mid‐puberty	PLEs	Females	−0.079**	−1.830**	0.377**	0.144**	27.625	**<.001**
Threat	Age at mid‐puberty	PLEs	Females	−0.127*	−0.320**	0.385**	0.041*	9.571	**0.038**
Threat	Slope at mid‐puberty	PLEs	Females	−0.030**	−2.259**	0.369**	0.069**	15.713	**0.002**
Dep‐IP	Age at mid‐puberty	PLEs	Females	−0.082*	−0.311**	0.275**	0.025*	8.468	**0.038**
Dep‐IP	Slope at mid‐puberty	PLEs	Females	−0.007	−2.403**	0.323**	0.018	5.212	0.121
Dep‐SE	Age at mid‐puberty	PLEs	Males	−0.189**	−0.274**	0.515**	0.052**	9.114	**0.012**
Dep‐SE	Slope at mid‐puberty	PLEs	Males	−0.053**	−1.068	0.469**	0.057	1.822	0.111
Threat	Age at mid‐puberty	PLEs	Males	−0.058	−0.294**	0.448**	0.017	3.699	0.192
Threat	Slope at mid‐puberty	PLEs	Males	−0.021**	−1.865**	0.429**	0.039*	8.414	**0.033**
Dep‐IP	Age at mid‐puberty	PLEs	Males	0.021	−0.287**	0.368**	−0.006	−1.629	0.425
Dep‐IP	Slope at mid‐puberty	PLEs	Males	−0.005	−1.820**	0.364**	0.010	2.583	0.192

*Note*: All values except those in the final column = unstandardised coefficients; pathways a and b correspond with those in Figure 1A; bolded values in final column = significant indirect pathways following FDR correction; * = *p* < 0.05; ** = *p* < 0.01; all models included age at baseline and the time from baseline to year 4 as covariates.

Abbreviations: Dep‐IP, Interpersonal deprivation; Dep‐SE, Socioeconomic deprivation; M, mediator; *X*, predictor variable; *Y*, dependent variable.

^a^ Direct effect refers to the total effect (*X* > *Y*) while controlling for the mediator.

Prior to FDR correction, several serial mediation effects from adversity to PLEs via altered puberty and steeper cortical volume decline were found in females (Supporting Information S1: Figure S5). Specifically, slower pubertal tempo and a more negative slope (i.e., steeper decline) in inferior and middle temporal, fusiform, lateral orbitofrontal and inferior parietal cortical volume mediated the relationship between dep‐SE and PLEs (explaining from 2.5% to 4.8% of the total effect). Mediation via slower pubertal tempo and steeper decline in inferior parietal and middle temporal cortical volume was also found for the association between threat and PLEs (1.8%–2.1% of the total effect). However, these pathways lost significance after FDR correction was applied across regions, and no serial mediation effects were found in males either before or after FDR correction (all *p* > 0.05).

### Secondary analyses

Repeating cortical volume models for thickness and surface area revealed no significant serial mediation effects after FDR correction, although several were found before, particularly for surface area. As with volume, these were limited to females and tended to implicate dep‐SE, threat and slower tempo (Supporting Information S1: Figures S6–S10).

### Sensitivity analyses

Most significant mediation effects were robust to additional covariates, with some exceptions. In males, the indirect effect of threat on PLEs via slower pubertal tempo lost significance when controlling for other adversity dimensions. In females, mediation of the threat‐PLE association by earlier pubertal timing lost significance when controlling for BMI, baseline PLEs and other adversity dimensions; the same was found for dep‐IP, except that the effect remained significant upon BMI inclusion (Supporting Information S1: Figure S11, panel D).

Though not preregistered, given the strong correlation between pubertal timing and tempo in our sample (*r* = 0.27 in males and *r* = 0.50 in females), we repeated our mediation models for each measure whilst controlling for the other. This rendered all mediation effects non‐significant (all *p* > 0.05).

## DISCUSSION

In this study, we investigated the role of individual differences in pubertal and GM development in pathways from adversity in childhood to PLEs in mid‐adolescence. We found that all adversity dimensions were associated with greater PLEs, in line with previous longitudinal work linking threat (Bell et al., 2019), and interpersonal (Croft et al., 2019) and socioeconomic (Croft et al., 2019; Newbury et al., 2023) deprivation with psychotic symptoms. Further, our study is the first to implicate puberty in these associations, with earlier timing and slower tempo partially mediating the majority of effects. In contrast to our hypothesis, results did not suggest that mediation effects could be further explained by puberty‐related alterations to GM development.

### Earlier and slower puberty mediate the relationship between adversity and PLEs, with effect sizes varying by sex and adversity dimension

Our prediction that altered pubertal timing would mediate associations between adversity and PLEs, but that the nature of mediation would vary by dimension, was partly supported. Although the proportion of variance explained by the mediators differed between models, all significant indirect effects were transmitted via earlier puberty, regardless of adversity dimension.

While no previous studies have explicitly tested a mediation pathway from childhood adversity to psychosis via pubertal timing, these findings align with research linking adversity (particularly threat) to earlier puberty (Colich et al., 2020), as well as evolutionary theories positing that puberty is brought forward in harsh contexts to maximise the chance of reproduction before death (Ellis, 2004). Notably, low SES (termed dep‐SE in the current framework) has also been associated with delayed puberty (Ellis et al., 2009). However, it has been suggested that poverty‐related delays in puberty may only occur in contexts of extreme energetic scarcity, where malnourishment may render successful reproduction unviable (Glass et al., 2022). As the United States is a high‐income country with low rates of underweight youth, the contribution of this phenomenon to our results was likely limited.

These pathways are also broadly consistent with models theorising that puberty interacts with pre‐existing vulnerabilities to confer psychosis risk (Barendse et al., 2023; Gomes et al., 2016), and extend them by suggesting that adversity‐related accelerations in pubertal timing might bring forward these interactions. One possibility is that earlier puberty exposes biological systems to these vulnerabilities before they have developed the necessary circuitry to cope with/compensate for them (Brooks‐Gunn et al., 1985; Ge & Natsuaki, 2009). For example, adversity‐related sensitisation of the HPA axis in childhood might lead to exaggerated puberty‐related increases in stress reactivity if the regions subserving stress regulation have not yet sufficiently matured to prevent this (Gomes et al., 2016; Roberts & Lopez‐Duran, 2019). Similarly, early puberty may afford synapses insufficient time to strengthen/mature prior to puberty‐related neuronal remodelling, resulting in inadequate synaptic protection in individuals with a predisposition for elevated pruning (Howes & Onwordi, 2023).

Our study is the first to examine pubertal tempo as a mediator in pathways from adversity to PLEs. We found that threat and dep‐SE (but not dep‐IP) predicted slower pubertal tempo, and in turn, greater PLEs, in males and females (except the pathway for dep‐SE in males). While these effects could reflect distinct mechanisms to those detected for pubertal timing, the significant correlation between timing and tempo (in both sexes), and the mutual loss of mediation effects when both pubertal metrics were included in models, suggest these pathways may be related. A possible explanation is that adversity‐related accelerations in pubertal onset might trigger compensatory mechanisms that cause subsequent pubertal development to slow, potentially to recalibrate to a normative developmental trajectory (Gunnar et al., 2019). The consequent extension of puberty could prolong elevations in plasticity/sensitivity (Schulz et al., 2009), lengthening the period for pre‐existing vulnerabilities to influence development and psychosis risk (Barendse et al., 2023). It could also provide longer for vulnerabilities to interact with inputs from the adolescent environment, consistent with models suggesting that early HPA axis sensitisation (Gomes et al., 2016) or aberrant programing of synaptic pruning (Howes & Onwordi, 2023) interact with adolescent stress to trigger psychotic symptoms.

Alternatively, as some previous studies (though not all (Hamlat et al., 2022; Miller et al., 2020; Suglia et al., 2020; Thijssen et al., 2022)) report *faster* pubertal tempo in adversity‐exposed youth (Ellis et al., 2011; Mendle et al., 2011), our results may partly reflect the approach used to model puberty. Whereas we adopted logistic growth curves, which assume that tempo peaks mid‐way through puberty, previous studies have tended to use linear approaches, which assume a uniform tempo throughout. A limitation of the latter is that, in right censored data (i.e., when data is missing for later timepoints), early accelerations in pubertal development may be captured but later decelerations missed, potentially producing faster tempo estimates in individuals with early timing (Beltz et al., 2014; Marceau et al., 2011). On the other hand, although logistic models better capture the non‐linear shape of pubertal development and are more robust to missing timepoints (Mendle et al., 2019), timing and tempo estimates are derived from the same curve inflection point, and are thus not fully independent of one another (Beltz et al., 2014; Marceau et al., 2011). Nevertheless, the separate mediation effects of pubertal timing and tempo observed warrant further investigation in longitudinal cohort studies.

While mediation pathways in males and females tended to be similar in nature (i.e., via earlier and slower puberty), they were consistently stronger in females. Previous studies investigating associations between pubertal variation and both adversity and psychopathology have likewise tended to find stronger effects in females (Milas et al., 2025; Oldehinkel & Bouma, 2011). Although the reasons for this are uncertain, it has been hypothesised that puberty may represent a more sensitive period in females than males (Sanacora et al., 2022). This is supported by work demonstrating greater pre‐to‐post puberty change in DNA methylation patterns in females, suggesting greater environmental responsiveness during this window (Thompson et al., 2018). The HPA axis may also be subject to divergent modulatory effects from oestrogen (excitatory) and testosterone (regulatory) (Roberts & Lopez‐Duran, 2019), potentially enabling better stress regulation in males after pubertal onset.

Notably, several mediation pathways for threat and dep‐IP (but not dep‐SE) lost significance when additional covariates were included, particularly the other adversity dimensions. Given cooccurrence between different forms of adversity, we cannot discount that the pathways for threat and dep‐IP may have been driven by dep‐SE‐related mechanisms. Another explanation may be that shared mechanisms (e.g., stress) underpin all three pathways to some degree, but that dep‐SE also confers risk via distinct mechanisms (e.g., exposure to endocrine disrupting chemicals (Demir et al., 2025; Nelson et al., 2012)).

### Mediation via puberty is not explained by change in grey matter structure

Our hypothesis that adversity would confer risk for PLEs via puberty‐related changes to GM was not supported. Serial mediation results suggested that the lack of full serial pathways was not attributable to associations between pubertal measures and GM, nor to intermediate mediation from adversity to GM via puberty. Indeed, these early‐chain effects were found for widespread regions (Supporting Information S1: Figures S5 and S10), consistent with prior work (MacSweeney et al., 2023). Instead, downstream paths were weaker, suggesting that while adversity may impact brain structure via puberty, these effects may not, in turn, transmit risk for PLEs.

Several previous studies examining pathways from puberty to other types of psychopathology via GM structure have also failed to find evidence for mediation (Kretzer et al., 2024). It may be that GM does not play a role in puberty‐related pathways to psychopathology, or that structural metrics examined in isolation are not sufficiently sensitive or comprehensive to capture the subtle or interdependent processes involved. Alternatively, as serial mediation effects were detected before FDR correction, it is possible that pathways were present in certain youth but were diluted in the wider sample. As discussed above, it is thought that pre‐existing vulnerabilities, embedded by various psychosis risk factors, interact with normal maturational processes such as puberty, setting development on an atypical course associated with psychosis risk (Barendse et al., 2023; Howes & Onwordi, 2023). These vulnerabilities might take different forms in different individuals (e.g., altered GM structure, dysregulated neurotransmitters, a primed immune system or sensitised HPA axis (Oliver et al., 2024)) and undergo distinct interactions with puberty to confer risk for psychosis via diverse pathways. Teasing apart these mechanisms represents a major challenge for future research.

### Limitations and future directions

Several limitations should be acknowledged. First, as our puberty modelling approach did not permit the presence of missing datapoints, we were only able to include participants with PDS data at every timepoint. This, and the random selection of 1 participant per family, resulted in a significantly reduced (albeit still large) sample relative to the wider cohort. Although this reduction is partly a reflection of the study's ongoing assessment schedule, demographic differences between included and excluded youth (e.g., higher income‐to‐needs ratio, a greater proportion of youth identifying as white, and less severe PLEs in the included sample) suggest that a degree of attrition bias may have been present. Accordingly, the observed pathways may be less generalisable to certain subgroups, such as non‐white youth or youth experiencing greater socioeconomic disadvantage. The underrepresentation of youth with greater symptom severity may also have influenced associations involving PLEs. However, because exclusion resulted from a range of factors (e.g., sibling exclusion, missed assessments, participant drop‐out, and the study's ongoing assessment schedule), it is difficult to determine the extent and direction of any attrition‐related bias.

Second, our study did not span the entirety of puberty for most participants, with many exceeding a PDS score of 1 at the first visit, and few reaching a score of 4 by the last. Although, relative to other approaches, logistic models mitigate censoring bias in this context (Mendle et al., 2019), they require observations around the curve's inflection point to estimate its shape. Participants transitioning through pubertal stages outside of the study window may, therefore, have contributed less to group estimates than those with better data coverage during these transitions.

Third, though we followed recommendations for creating adversity dimension composites (Berman et al., 2022), tools designed specifically to measure deprivation and threat may more closely reflect the hypothesised constructs (Cohodes et al., 2023). It may also be valuable to examine whether similar findings emerge for adversity dimensions derived using data‐driven (i.e., bottom‐up) approaches (e.g., see Brieant et al., 2023), which may better capture naturally occurring patterns of adversity. Additionally, given that adversity was measured at baseline, we were unable to infer exactly when these exposures occurred. As the timing of adversity may be key in determining its biological and clinical impact (Gee & Casey, 2015), developing strategies that isolate time‐varying effects will be important for informing targeted interventions for sensitive periods of development.

Fourth, the whole‐brain approach adopted in our neuroimaging analyses required correction across a large number of statistical models. While this was necessary to reduce the likelihood of false‐positive findings, it may have reduced sensitivity to detect small regional effects. Future studies may benefit from evaluating the regions implicated in our study (i.e., prior to FDR correction) using more targeted, hypothesis‐driven approaches.

It is also worth noting that though we chose to aggregate constituent indicators of socioeconomic disadvantage within the dep‐SE dimension based on prior work demonstrating similar neuroanatomical associations (e.g., Thomas et al., 2025), other research has revealed evidence for distinct or interacting associations between these indicators and both brain structure (Rakesh et al., 2022) and psychopathology (Larson, Moussa‐Tooks, et al., 2025). Subsequent work should therefore aim to identify both shared and distinct pathways linking different components of socioeconomic deprivation to psychosis risk.

Future studies investigating the role of sex hormones (including within‐subject variability) in these pathways could help decipher the cellular mechanisms underpinning our findings. Additionally, incorporating other biological domains via additional moderator paths, stratified biomarker‐derived subgroups, or multivariate approaches, may illuminate heterogeneous pathways that might have been masked in our study. Investigating the role of social factors related to pubertal development (e.g., changing role expectations) will also be important (Vijayakumar et al., 2024).

### Conclusion

Our findings contribute to growing evidence linking adversity to psychosis risk, and extend the literature by demonstrating an indirect pathway via altered pubertal development. The strength of mediation effects varied by sex and adversity dimension, although the nature of pathways tended to be similar. We did not find strong evidence that mediation via puberty was due to its influence on GM development. To contextualise our findings and disentangle heterogeneity in these pathways, future studies should use alternative modelling techniques to incorporate data on sex hormones and additional developmental processes.

## AUTHOR CONTRIBUTIONS

**Megan Thomas.** Conceptualization, Methodology, Formal Analysis, Visualization, Writing — Original Draft Preparation. **Sarah Whittle.** Supervision, Conceptualization, Methodology, Writing — Review & Editing. **Elizabeth M. Haris.** Writing – Review & Editing. **Vanessa L. Cropley.** Supervision, Conceptualization, Methodology, Writing – Review & Editing.

## CONFLICT OF INTEREST STATEMENT

The authors declare no conflicts of interest.

## ETHICAL CONSIDERATIONS

The ABCD study was approved by a central Institutional Review Board at the University of California, San Diego (IRB #160091, approved September 13, 2016). Written parental consent and participant assent were obtained at all sites.

## AI STATEMENT

The authors declare that they have not used AI in any capacity that would require disclosure in accordance with Wiley's AI Guidelines, nor in any capacity that would reasonably require discloser for editors, reviewers and readers to properly evaluate their research or manuscript. The authors confirm that AI has not been used for purposes including but NOT limited to drafting and editing, to generate substantial text or restructure arguments, nor in the research methodology. The authors take full responsibility for the accuracy of this statement.

## Supporting information

Supporting Information S1

## Data Availability

The data that support the findings of this study are available via the NIMH Data Archive (https://nda.nih.gov/abcd). Access is subject to an approved data use agreement.
