# Supplementary material for: Childhood adversity and psychotic‐like experiences in adolescence: Investigating a pathway via disrupted pubertal and grey matter development
Source: JCPP Adv. 2026 Aug 18:e70159. Online ahead of print. doi: 10.1002/jcv2.70159 (PMC13487410; doi:10.1002/jcv2.70159)
Supplement: Supplementary file 1 [file JCV2-9999-e70159-s001.docx]

**Childhood adversity and psychotic-like experiences in adolescence: investigating a pathway via disrupted pubertal and grey matter development**

**Supporting Information**

**Table of contents**

**1.** **Appendices**	**2**

Appendix S1. Composition of childhood adversity measures	2

Appendix S2. Exclusions and handling of missing data	3

**2.** **Supporting Figures**	**4**

Figure S1. Regional growth trajectories (cortical volume) in males	4

Figure S2. Regional growth trajectories (cortical volume) in females	5

Figure S3. Regional growth trajectories (subcortical volume) in males	6

Figure S4. Regional growth trajectories (subcortical volume) in females	7

Figure S5. Serial mediation models (primary)	8

Figure S6. Regional growth trajectories (cortical thickness) in males	14

Figure S7. Regional growth trajectories (cortical thickness) in females	15

Figure S8. Regional growth trajectories (cortical surface area) in males	16

Figure S9. Regional growth trajectories (cortical surface area) in females	17

Figure S10. Serial mediation models (secondary)	18

Figure S11. Sensitivity analyses: mediation model paths with and without additional covariates	32

**3. Supporting Tables**	**33**

Table S1. Included and excluded sample characteristics at baseline in males	33

Table S2. Included and excluded sample characteristics at baseline in females	34

Table S3. Pearson’s correlations among non-brain variables in males	35

Table S4. Pearson’s correlations among non-brain variables in females	35

Table S5. Proportion of data imputed for study measures	36

Table S6. Brain region abbreviations and full names	37

**4. References**	**38**

**Appendix S1. Composition of childhood adversity dimensions**

The measures comprising each composite score were chosen to capture the dimensions of deprivation and threat operationalised by dimensional model of adversity and psychopathology (DMAP) (McLaughlin et al., 2014), but with deprivation parsed into interpersonal and socioeconomic subtypes. All measures contributing to these scores were reverse scored where appropriate, such that higher values equated to greater adversity. They were then standardised (using z-scoring) and averaged within each dimension subtype (e.g. material deprivation), then averaged within each dimension (e.g. socioeconomic deprivation) to create three overarching composite scores. The measures comprising each are outlined below.

**Threat**

Interpersonal trauma: 12 items of the ABCD Parent Diagnostic Interview for DSM-5 (KSADS) Traumatic Events scale that relate to interpersonal trauma. Items that measure exposure to natural disasters, fires, car accidents, other accidents and the death of a loved one were excluded, as non-interpersonal forms of trauma are not typically included in the threat dimension and may have different developmental impacts to those hypothesised in the DMAP (Berman et al., 2022; Bounoua et al., 2024; Forbes et al., 2014; McLaughlin & Lambert, 2017).

Neighbourhood crime: Neighbourhood crime rates derived using participants’ residential address and data from “Uniform Crime Reports”.

Family conflict: youth and parent scores on the 9-item Family Conflict subscale of the Family Environment Scale (MOOS, 1990). For each item, endorsement by either the youth or parent resulted in that item being recorded as present, in line with recommendations (Berman et al., 2022).

Bullying: youth and parent responses on the ABCD Parent/Youth Diagnostic Interviews for DSM-5 Background Items Full (KSADS-5): “Does your child / do you have any problems with bullying at school or in your neighborhood?”. As for family conflict, endorsement by either the youth or parent was considered overall endorsement.

**Interpersonal deprivation**

Caregiver neglect: The 5-item youth-rated Parental Monitoring Scale (Karoly et al., 2016; Zucker et al., 2018) and 5 items taken from the Child Report of Parenting Behaviour Inventory (CRPBI) (Barber, 1997; Schaefer, 1965) assessing caregiver acceptance. As CRPBI items relate to individual caregivers, the average score across all caregivers was used when more than one was available.

**Socioeconomic deprivation**

Material deprivation: The 7-item parent-rated Financial Adversity Questionnaire (PRFQ) (Diemer et al., 2013) and family income-to-needs ratio (combined household income divided by the federal poverty level for a family of the same size).

Low parental education: The highest level of schooling (reverse-scored) reached by the primary caregiver, or the average level across all caregivers when available (from 0=never attended/kindergarten to 21=doctoral degree).

Neighbourhood disadvantage: The weighted sum of the Area Deprivation Index (ADI) (Singh, 2003), computed using participants’ residential address at baseline.

**Appendix S2. Exclusions and handling of missing data**

The ABCD dataset contains 11,868 participants at baseline. Of these, one participant per family was selected at random (N=9,807) and all other siblings were excluded (N=2,061).

Within-subject mean replacement of missing data was performed for study measures that encompassed several items. Specifically, if a participant had <=30% of items missing for a particular measure, missing items would be replaced with the mean of the remaining (complete) items. Total scores for each measure were then calculated.

Following mean-replacement, participants without PDS scores at all timepoints were excluded (N=6,345). In our remaining sample (N=3,462), all data still missing for PLE scores, mean puberty scores, and measures contributing to adversity constructs (e.g. total PMQ) were imputed using multiple imputation by chained equations (MICE). Using the “mice” R package (van Buuren & Groothuis-Oudshoorn, 2011), we used predictive mean matching to generate 50 datasets with 50 iterations per imputation, which were then combined using the “merge_imputations” function in the “sjmisc” R package (see Table S5 for proportion of study measures imputed).

Given that linear mixed models are able to estimate random effects and slopes in the presence of missing data, and using imputation and LMMs together can cause model instability (Twisk et al., 2013), we did not impute missing data for imaging metrics (i.e. regional cortical volume, thickness or surface area, and regional subcortical volume). As two datapoints per subject are required to fit random slopes, and only imaging data passing the ABCD Data and Informatics Centre (DAIC) quality checking procedure was included in our analyses, this reduced our sample size for analyses including imaging metrics from N=3,462 to N=3,159.

**Figure S1. Cortical volume in mm^3^ (y axis) by age (x axis) in males.**

Black lines = average regional volume trajectory; purple lines = raw subject-specific trajectories; volume measurements pertain to the sum of both hemispheres; see Table S6 for full region names.

**Figure S2. Cortical volume in mm^3^ (y axis) by age (x axis) in females.**

Black lines = average regional volume trajectory; orange lines = raw subject-specific trajectories; volume measurements pertain to the sum of both hemispheres; see Table S6 for full region names.

**Figure S3. Subcortical volume in mm^3^ (y axis) by age (x axis) in males.**

Black lines = average regional volume trajectory; purple lines = raw subject-specific trajectories; volume measurements pertain to the sum of both hemispheres; see Table S6 for full region names.

**Figure S4. Subcortical volume in mm^3^ (y axis) by age (x axis) in females.**

Black lines = average regional volume trajectory; orange lines = raw subject-specific trajectories; volume measurements pertain to the sum of both hemispheres; see Table S6 for full region names.

**Figure S5. Serial mediation models**

The following charts for models 1-7 display effect sizes and confidence intervals for each serial mediation model conducted for grey matter volume (GMV) (see Figure 1B). **Path a_1_d_1_** = effect of adversity on grey matter slope via pubertal timing/tempo (calculated as the product of the a_1_ and d_1_ paths); **Path d_1_b_2_** = effect of pubertal timing/tempo on PLEs via grey matter slope, corrected for adversity (i.e. d_1_ x b_2_); **Path a_1_d_1_b_2_** = effect of adversity on PLEs via puberty *and* grey matter slope (i.e. a_1_ x d_1_ x b_2_); IRR = incidence rate ratio; see Table S6 for full region names. Dashed vertical lines represent no effect. For path a_1_d_1_b_2_, significant serial mediation effects (before FDR correction) are shown in black. To demonstrate the nature of intermediary mediation effects, paths a_1_d_1_ and d_1_b_2_ are coloured in blue (-) or pink (+) depending on the direction of the a_1_ (for path a_1_d_1_) and d_1_ (for path d_1_b_2_) effect, respectively. For example, in Model 1, blue lines below 0 for path a_1_d_1_ indicate that dep-SE predicted a more negative GMV slope via *lower* age at mid-puberty. All models included age at baseline, time from baseline to year 4, and the random intercept term for the corresponding brain region, as covariates. Due to variance limits in Mplus, random intercepts for cortical volume were divided by 100 prior to serial mediation analyses.

**Model 1: dep-SE > age at mid-puberty > GMV slope > PLEs (females)**

**Model 2: dep-SE > slope at mid-puberty > GMV slope > PLEs (females)**

**Model 3: threat > age at mid-puberty > GMV slope > PLEs (females)**

**Model 4: threat > slope at mid-puberty > GMV slope > PLEs (females)**

**Model 5: dep-IP > age at mid-puberty > GMV slope > PLEs (females)**

**Model 6: dep-SE > age at mid-puberty > GMV slope > PLEs (males)**

**Model 7: threat > slope at mid-puberty > GMV slope > PLEs (males)**

**Figure S6. Cortical thickness in mm (y axis) by age (x axis) in males.**

Black lines = average regional thickness trajectory; purple lines = raw subject-specific trajectories; thickness measurements pertain to the mean of both hemispheres; see Table S6 for full region names.

**Figure S7. Cortical thickness in mm (y axis) by age (x axis) in females.**

Black lines = average regional thickness trajectory; orange lines = raw subject-specific trajectories; thickness measurements pertain to the mean of both hemispheres; see Table S6 for full region names.

**Figure S8. Cortical surface area in mm^2^ (y axis) by age (x axis) in males.**

Black lines = average regional surface area trajectory; purple lines = raw subject-specific trajectories; surface area measurements pertain to the sum of both hemispheres; see Table S6 for full region names.

**Figure S9. Cortical surface area in mm^2^ (y axis) by age (x axis) in females.**

Black lines = average regional surface area trajectory; orange lines = raw subject-specific trajectories; surface area measurements pertain to the sum of both hemispheres; see Table S6 for full region names.

**Figure S10. Serial mediation models**

The following charts for models 8-13 (cortical thickness [CT]) and 14-21 (cortical surface area [SA]) display effect sizes and confidence intervals for each secondary serial mediation model conducted (see Figure 1B). **Path a_1_d_1_** = effect of adversity on CT/SA slope via pubertal timing/tempo (calculated as the product of the a_1_ and d_1_ paths); **Path d_1_b_2_** = effect of pubertal timing/tempo on PLEs via CT/SA slope, corrected for adversity (i.e. d_1_ x b_2_); **Path a_1_d_1_b_2_** = effect of adversity on PLEs via puberty *and* CT/SA slope (i.e. a_1_ x d_1_ x b_2_); IRR = incidence rate ratio; see Table S6 for full region names. Dashed vertical lines represent no effect. For path a_1_d_1_b_2_, significant serial mediation effects (before FDR correction) are shown in black. To demonstrate the nature of intermediary mediation effects, paths a_1_d_1_ and d_1_b_2_ are coloured in blue (-) or pink (+) depending on the direction of the a_1_ (for path a_1_d_1_) and d_1_ (for path d_1_b_2_) effect, respectively. All models included age at baseline, time from baseline to year 4, and the random intercept term for the corresponding brain region, as covariates. Due to variance limits in Mplus, random intercepts for surface area were divided by 100, and random slopes for cortical thickness were multiplied by 1000, prior to serial mediation analyses.

**Model 8: dep-SE > age at mid-puberty > CT slope > PLEs (females)**

**Model 9: dep-SE > slope at mid-puberty > CT slope > PLEs (females)**

**Model 10: threat > age at mid-puberty > CT slope > PLEs (females)**

**Model 11: threat > slope at mid-puberty > CT slope > PLEs (females)**

**Model 12: dep-IP > age at mid-puberty > CT slope > PLEs (females)**

**Model 13: dep-SE > age at mid-puberty > CT slope > PLEs (males)**

**Model 14: threat > slope at mid-puberty > CT slope > PLEs (males)**

**Model 15: dep-SE > age at mid-puberty > SA slope > PLEs (females)**

**Model 16: dep-SE > slope at mid-puberty > SA slope > PLEs (females)**

**Model 17: threat > age at mid-puberty > SA slope > PLEs (females)**

**Model 18: threat > slope at mid-puberty > SA slope > PLEs (females)**

**Model 19: dep-IP > age at mid-puberty > SA slope > PLEs (females)**

**Model 20: dep-SE > age at mid-puberty > SA slope > PLEs (males)**

**Model 21: threat > slope at mid-puberty > SA slope > PLEs (males)**

**Figure S11. Sensitivity analyses: mediation model paths with and without additional covariates.**

A

B

C

D

E

Each panel displays the sex specific (orange = females; purple = males) effect sizes and confidence intervals for paths within our mediation models (see Figure 1A), before (in colour) and after (in grey) including additional covariates (BMI, PLEs at baseline, and the two other adversity dimensions). **Panel A** = incidence rate ratios (IRRs) for total effects (adversity > PLEs); **Panel B** = standardised coefficients for path a (adversity > puberty); **Panel C** = IRRs for path b (puberty > PLEs, controlling for adversity); **Panel D** = IRRs for indirect effects (adversity > puberty > PLEs); **Panel E** = IRRs for direct effects (adversity > PLEs, controlling for puberty). In A, grey dashed lines at x=0 represent no effect. In B, C and D, grey dashed lines at x=1 represent no effect. Effects above and below these values indicate positive and negative effects, respectively. All paths are adjusted for age at baseline, and time from baseline to 4-year follow up.

Table S1. Included and excluded sample characteristics at baseline in males.

|  | **Included**  **(N=1,862)** | | **Excluded**  **(N=4,326)** | |  |  |  |
| --- | --- | --- | --- | --- | --- | --- | --- |
|  | **M** | **SD** | **M** | **SD** | **Test statistic** | **p** | **d** |
| **Age (months)** | 119.234 | 7.456 | 119.087 | 7.531 | *t(*3559) = -0.707 | .480 | -0.02 |
| **Income-to-needs ratio*** | 3.897 | 2.378 | 3.542 | 2.407 | *t(*3362) = -5.118 | <.001 | -.148 |
| **BMI** (**kg/m²)** | 18.717 | 4.04 | 18.731 | 4.177 | *t(*3637) = 0.126 | .899 | .003 |
| **PQ-BC score** | 6.22 | 10.481 | 6.584 | 10.803 | *t(*3625) = 1.241 | .215 | .034 |
| **PDS score** | 1.42 | 0.361 | 1.455 | 0.39 | *t(*3835) = 3.409 | .001 | .094 |
|  | **N** | **%** | **N** | **%** | **Test statistic** | **p** | **v** |
| **Race** |  |  |  |  |  |  |  |
| White | 1518 | 81.5 | 3121 | 72.1 | *X²(*1, N=6188) = 60.53 | <.001 | .099 |
| Black | 248 | 13.3 | 1020 | 23.6 | *X²(*1, N=6188) = 83.47 | <.001 | .116 |
| Asian | 119 | 6.4 | 257 | 5.9 | *X²(*1, N=6188) = 0.39 | .534 | .008 |
| Native American | 61 | 3.3 | 162 | 3.7 | *X²(*1, N=6188) = 0.69 | .405 | .011 |
| Pacific Islander | 6 | 0.3 | 26 | 0.6 | *X²(*1, N=6188) = 1.46 | .227 | .015 |
| Other | 115 | 6.2 | 303 | 7 | *X²(*1, N=6188) = 1.29 | .256 | .014 |
| None given | 19 | 1 | 72 | 1.7 | *X²(*1, N=6188) = 3.29 | .070 | .023 |

*<1 = below poverty threshold; BMI = body mass index; d = Cohen’s d; M = mean; SD = standard deviation; PDS = Pubertal Development Scale; PQ-BC = youth-rated Prodromal Questionnaire–Brief Child Version; v = Cramer’s v.

Table S2. Included and excluded sample characteristics at baseline in females.

|  | **Included**  **(N=1,600)** | | **Excluded**  **(N=4,077)** | |  |  |  |
| --- | --- | --- | --- | --- | --- | --- | --- |
|  | **M** | **SD** | **M** | **SD** | **Test statistic** | **p** | **d** |
| **Age (months)** | 119.266 | 7.246 | 118.631 | 7.563 | *t(*3041) = -2.932 | .003 | -.086 |
| **Income-to-needs ratio*** | 3.955 | 2.417 | 3.512 | 2.419 | *t(*2739) = -5.941 | <.001 | -.183 |
| **BMI** (**kg/m²)** | 18.614 | 4.069 | 19.072 | 4.471 | *t(*3191) = 3.712 | <.001 | .107 |
| **PQ-BC score** | 5.699 | 9.535 | 6.316 | 10.845 | *t(*3300) = 2.108 | .035 | .060 |
| **PDS score** | 1.718 | 0.494 | 1.798 | 0.537 | *t(*3200) = 5.329 | <.001 | .155 |
|  | **N** | **%** | **N** | **%** | **Test statistic** | **p** | **v** |
| **Race** |  |  |  |  |  |  |  |
| White | 1270 | 79.4 | 2886 | 70.8 | *X²(*1, N=5677) = 42.77 | <.001 | .087 |
| Black | 258 | 16.1 | 991 | 24.3 | *X²(*1, N=5677) = 44.35 | <.001 | .088 |
| Asian | 118 | 7.4 | 257 | 6.3 | *X²(*1, N=5677) = 1.97 | .161 | .019 |
| Native American | 69 | 4.3 | 138 | 3.4 | *X²(*1, N=5677) = 2.56 | .110 | .021 |
| Pacific Islander | 6 | 0.4 | 15 | 0.4 | *X²(*1, N=5677) = 0 | 1.000 | .000 |
| Other | 85 | 5.3 | 297 | 7.3 | *X²(*1, N=5677) = 6.81 | .009 | .035 |
| None given | 19 | 1.2 | 76 | 1.9 | *X²(*1, N=5677) = 2.8 | .094 | .022 |

*<1 = below poverty threshold; BMI = body mass index; d = Cohen’s d; M = mean; SD = standard deviation; PDS = Pubertal Development Scale; PQ-BC = youth-rated Prodromal Questionnaire–Brief Child Version; v = Cramer’s v.

**Table S3. Pearson’s correlations among non-brain variables in males.**

|  | ageBL | ageY4 | agediff | bmi | ppsBL | ppsY4 | threat | Dep-SE | Dep-IP | timing | tempo |
| --- | --- | --- | --- | --- | --- | --- | --- | --- | --- | --- | --- |
| ageBL | 1.00*** |  |  |  |  |  |  |  |  |  |  |
| ageY4 | 0.95*** | 1.00*** |  |  |  |  |  |  |  |  |  |
| agediff | 0.10*** | 0.40*** | 1.00*** |  |  |  |  |  |  |  |  |
| bmi | 0.10*** | 0.08*** | -0.04 | 1.00*** |  |  |  |  |  |  |  |
| ppsBL | -0.11*** | -0.12*** | -0.06* | 0.05* | 1.00*** |  |  |  |  |  |  |
| ppsY4 | -0.06* | -0.07** | -0.07** | 0.09*** | 0.28*** | 1.00*** |  |  |  |  |  |
| threat | -0.10*** | -0.10*** | -0.01 | 0.03 | 0.20*** | 0.09*** | 1.00*** |  |  |  |  |
| Dep-SE | -0.04 | -0.06** | -0.08*** | 0.21*** | 0.16*** | 0.13*** | 0.14*** | 1.00*** |  |  |  |
| Dep-IP | -0.07** | -0.07** | -0.04 | 0.03 | 0.20*** | 0.12*** | 0.16*** | 0.13*** | 1.00*** |  |  |
| timing | 0.14*** | 0.16*** | 0.09*** | -0.21*** | -0.09*** | -0.09*** | -0.05* | -0.13*** | 0.01 | 1.00*** |  |
| tempo | 0.07** | 0.06** | 0.00 | -0.21*** | -0.13*** | -0.07** | -0.10*** | -0.26*** | -0.04 | 0.27*** | 1.00*** |

** = p<.05; ** = p<.01*

**Table S4. Pearson’s correlations between brain and non-brain variables in females.**

|  | ageBL | ageY4 | agediff | bmi | ppsBL | ppsY4 | threat | Dep-SE | Dep-IP | timing | tempo |
| --- | --- | --- | --- | --- | --- | --- | --- | --- | --- | --- | --- |
| ageBL | 1.00*** |  |  |  |  |  |  |  |  |  |  |
| ageY4 | 0.95*** | 1.00*** |  |  |  |  |  |  |  |  |  |
| agediff | 0.10*** | 0.40*** | 1.00*** |  |  |  |  |  |  |  |  |
| bmi | 0.10*** | 0.08** | -0.06* | 1.00*** |  |  |  |  |  |  |  |
| ppsBL | -0.05* | -0.06* | -0.03 | 0.06* | 1.00*** |  |  |  |  |  |  |
| ppsY4 | -0.02 | -0.04 | -0.05* | 0.06* | 0.21*** | 1.00*** |  |  |  |  |  |
| threat | -0.04 | -0.02 | 0.05* | 0.07** | 0.20*** | 0.09*** | 1.00*** |  |  |  |  |
| Dep-SE | -0.07** | -0.09*** | -0.09*** | 0.28*** | 0.16*** | 0.15*** | 0.13*** | 1.00*** |  |  |  |
| Dep-IP | -0.08** | -0.08** | -0.01 | 0.00 | 0.17*** | 0.11*** | 0.15*** | 0.09*** | 1.00*** |  |  |
| timing | 0.10*** | 0.11*** | 0.06* | -0.35*** | -0.12*** | -0.16*** | -0.06* | -0.21*** | -0.07** | 1.00*** |  |
| tempo | 0.01 | 0.02 | 0.03 | -0.29*** | -0.15*** | -0.15*** | -0.12*** | -0.34*** | -0.05 | 0.50*** | 1.00*** |

** = p<.05; ** = p<.01*

**Table S5. Percentage of data imputed for study variables.**

| **Variable** | **N imputed (%)** |
| --- | --- |
| BMI (BL) | 2 (0.1) |
| Total PFRQ (BL) | 5 (0.1) |
| INR (BL) | 260 (7.5) |
| Total PMQ (BL) | 5 (0.1) |
| Total parent CRPBI (BL) | 9 (0.3) |
| Area crime rates (BL) | 130 (3.8) |
| Total traumatic events (BL) | 132 (3.8) |
| Parent education (BL) | 5 (0.1) |
| ADI score (BL) | 130 (3.8) |
| PQ-BC score (BL) | 2 (0.1) |
| PQ-BC score (Y4) | 6 (0.2) |

BL = baseline; Y4 = 4 year follow up; NB: age, sex, pubertal timing and tempo, grey matter volume intercepts and slopes for all regions, and any other childhood adversity measures without missing data, also contributed to MICE imputation of these variables.

**Table S6. Brain region abbreviations and full names.**

| **Abbreviation** | **Full name** |
| --- | --- |
| bankssts | Banks of the superior temporal sulcus |
| cdacate | Caudal anterior cingulate |
| cdmdfr | Caudal middle frontal |
| cuneus | Cuneus |
| ehinal | Entorhinal |
| fusiform | Fusiform |
| ifpl | Inferior parietal |
| iftm | Inferior temporal |
| ihcate | Isthmus cingulate |
| locc | Lateral occipital |
| lobfr | Lateral orbitofrontal |
| lingual | Lingual |
| mobfr | Medial orbitofrontal |
| mdtm | Middle temporal |
| parahpal | Parahippocampal |
| paracn | Paracentral |
| parsopc | Pars opercularis |
| parsobis | Pars orbitalis |
| parstgris | Pars triangularis |
| pericc | Pericalcarine |
| postcn | Postcentral |
| ptcate | Posterior cingulate |
| precn | Precentral |
| pc | Precuneus |
| rracate | Rostral anterior cingulate |
| rrmdfr | Rostral middle frontal |
| sufr | Superior frontal |
| supl | Superior parietal |
| sutm | Superior temporal |
| sm | Supramarginal |
| frpole | Frontal pole |
| tmpole | Temporal pole |
| trvtm | Transverse temporal |
| insula | Insula |
| tp | Thalamus proper |
| caudate | Caudate |
| putamen | Putamen |
| pallidum | Pallidum |
| hpus | Hippocampus |
| amygdala | Amygdala |
| aa | Accumbens area |

**References**

Barber, B. K. (1997). Introduction: Adolescent Socialization in Context-the Role of Connection, Regulation, and Autonomy in the Family. *Journal of Adolescent Research*, *12*(1), 5–11. https://doi.org/10.1177/0743554897121002

Berman, I. S., McLaughlin, K. A., Tottenham, N., Godfrey, K., Seeman, T., Loucks, E., Suomi, S., Danese, A., & Sheridan, M. A. (2022). Measuring early life adversity: A dimensional approach. *Development and Psychopathology*, *34*(2), 499–511. https://doi.org/DOI: 10.1017/S0954579421001826

Bounoua, N., Joseph, J. E., Adams, Z. W., Crum, K. I., Sege, C. T., McTeague, L. M., Hajcak, G., Halliday, C. A., & Danielson, C. K. (2024). Interpersonal violence moderates sustained-transient threat co-activation in the vmPFC and amygdala in a community sample of youth. *Development and Psychopathology*, 1–10. https://doi.org/DOI: 10.1017/S0954579424001743

Diemer, M. A., Mistry, R. S., Wadsworth, M. E., López, I., & Reimers, F. (2013). Best Practices in Conceptualizing and Measuring Social Class in Psychological Research. *Analyses of Social Issues and Public Policy*, *13*(1), 77–113. https://doi.org/https://doi.org/10.1111/asap.12001

Forbes, D., Lockwood, E., Phelps, A., Wade, D., Creamer, M., Bryant, R. A., McFarlane, A., Silove, D., Rees, S., Chapman, C., Slade, T., Mills, K., Teesson, M., & O’Donnell, M. (2014). Trauma at the hands of another: distinguishing PTSD patterns following intimate and nonintimate interpersonal and noninterpersonal trauma in a nationally representative sample. *The Journal of Clinical Psychiatry*, *75*(2), 147–153. https://doi.org/10.4088/JCP.13m08374

Karoly, H. C., Callahan, T., Schmiege, S. J., & Feldstein Ewing, S. W. (2016). Evaluating the Hispanic Paradox in the Context of Adolescent Risky Sexual Behavior: The Role of Parent Monitoring. *Journal of Pediatric Psychology*, *41*(4), 429–440. https://doi.org/10.1093/jpepsy/jsv039

McLaughlin, K. A., & Lambert, H. K. (2017). Child Trauma Exposure and Psychopathology: Mechanisms of Risk and Resilience. *Current Opinion in Psychology*, *14*, 29–34. https://doi.org/10.1016/j.copsyc.2016.10.004

McLaughlin, K. A., Sheridan, M. A., & Lambert, H. K. (2014). Childhood adversity and neural development: deprivation and threat as distinct dimensions of early experience. *Neuroscience and Biobehavioral Reviews*, *47*, 578–591. https://doi.org/10.1016/j.neubiorev.2014.10.012

MOOS, R. H. (1990). Conceptual and Empirical Approaches to Developing Family-Based Assessment Procedures: Resolving the Case of the Family Environment Scale. *Family Process*, *29*(2), 199–208. https://doi.org/https://doi.org/10.1111/j.1545-5300.1990.00199.x

Schaefer, E. S. (1965). A configurational analysis of children’s reports of parent behavior. In *Journal of Consulting Psychology* (Vol. 29, Issue 6, pp. 552–557). American Psychological Association. https://doi.org/10.1037/h0022702

Singh, G. K. (2003). Area Deprivation and Widening Inequalities in US Mortality, 1969–1998. *American Journal of Public Health*, *93*(7), 1137–1143. https://doi.org/10.2105/AJPH.93.7.1137

Twisk, J., de Boer, M., de Vente, W., & Heymans, M. (2013). Multiple imputation of missing values was not necessary before performing a longitudinal mixed-model analysis. *Journal of Clinical Epidemiology*, *66*(9), 1022–1028. https://doi.org/10.1016/j.jclinepi.2013.03.017

van Buuren, S., & Groothuis-Oudshoorn, K. (2011). mice: Multivariate Imputation by Chained Equations in R. *Journal of Statistical Software*, *45*(3 SE-Articles), 1–67. https://doi.org/10.18637/jss.v045.i03

Zucker, R. A., Gonzalez, R., Feldstein Ewing, S. W., Paulus, M. P., Arroyo, J., Fuligni, A., Morris, A. S., Sanchez, M., & Wills, T. (2018). Assessment of culture and environment in the Adolescent Brain and Cognitive Development Study: Rationale, description of measures, and early data. *Developmental Cognitive Neuroscience*, *32*, 107–120. https://doi.org/10.1016/j.dcn.2018.03.004
